# Reluctance of women of lower socio-economic status to use maternal healthcare services – Does only cost matter?

**DOI:** 10.1371/journal.pone.0239597

**Published:** 2020-09-29

**Authors:** Sanzida Akhter, Gouranga Lal Dasvarma, Udoy Saikia

**Affiliations:** 1 Department of Women and Gender Studies, University of Dhaka, Dhaka, Bangladesh; 2 College of Humanities, Arts and Social Sciences, Flinders University, Adelaide, Australia; USC Keck School of Medicine, Institute for Global Health, UNITED STATES

## Abstract

In this paper we examine whether it is just the financial cost of maternal healthcare that prevents poor women from utilising free or low-cost government provided healthcare in Dhaka, Bangladesh, or there are other factors at play, in conjunction with poverty. To answer this question, we analyse the perceptions and experiences about the use of maternal health care for childbirth by a group of women residing in poor and lower socio-economic households in Dhaka. Data for this study were collected through in-depth interviews of 34 such women who have already had a child or had become pregnant at least once in the preceding five years. The findings of our analysis suggest that these women have a deeply rooted fear of medical intervention in childbirth for several perceived and practical reasons, including the fear of having to make undocumented payments, unfamiliarity with institutional processes, lack of social and family network support within their neighbourhood, concept of honour and shame [*sharam]*, a culture of silence and inadequate spousal communication on health issues. As a result, even though low-cost health care facilities may be within their reach in terms of physical distance and affordable in terms of financial cost these women and their families are unwilling to deliver their babies at such health facilities. Therefore, in order to allay their perceived fear of hospital-based childbirth, one needs to consider factors other than financial cost and physical distance, and provide these women with factual information and culturally sensitive counselling.

## 1. Introduction

Bangladesh has expanded its maternal health service by setting up low cost government and non-government hospitals of different levels all over the country, so that women can reach them conveniently, and free of charge or at low cost. Consequently, the country has achieved a remarkable reduction in maternal mortality ratio, reduction of fertility and expansion of the coverage of antenatal care (ANC) [1]. Since the 1990s, various initiatives have been taken by government and non-government organisations in Bangladesh to help poor women access appropriate maternal healthcare, including free or low-cost maternal healthcare. Yet, as of 2017, only 26.4% of the poorest women of urban Bangladesh delivered their babies at health facilities and 27.8% received assistance from skilled birth attendants during delivery, compared to 78.3% and 82.6% respectively of the richest women delivering at health facilities and receiving assistance from skilled birth attendants at childbirth [2]. These findings indicate that cost might not be the sole reason for such low utilisation of maternal healthcare by poor women of Bangladesh.

This paper aims to find out, with reference to women of low socio-economic households in the Bangladesh capital, Dhaka, whether only costs drive their low utilisation of maternal healthcare, particularly at childbirth or are there other factors in conjunction with poverty that also play a role in this phenomenon. To fulfil this aim, this study examines whether the socio-economic status of these women, together with their own experience or the experience of their friends and neighbours influence their healthcare behaviour during and after childbirth. It may be noted that, in this context cost refers to the fees that one has to pay to visit these health facilities, however small those fees may be.

Based on primary data, collected from dwellers of informal settlements (or slums) in a major urban area of a developing country, this study provides an insight into the roles of financial cost or geographical proximity versus women’s perception, beliefs and cultural inhibitions in the low uptake of maternal healthcare and childbirth in modern institutional settings. This research contributes to scientific knowledge in the field of maternal health which can be replicated with appropriate modifications in other situations.

The findings of the study are based on a qualitative analysis of data collected by interviewing women in living two informal urban settlements of Dhaka, Bangladesh. Details about the sampling and data collection are given in the section on Materials and Methods. This paper is extracted from the PhD dissertation of the first author written under the supervision of the co-authors at Flinders University, Australia. This research is conducted with due observance of research ethics involving human subjects. It was approved in Australia by the Social and Behavioural Research Ethics Committee, Flinders University. In Bangladesh, necessary permission was obtained from the health facilities where some of the participants were interviewed and signed informed consent was obtained from all the participants before they were interviewed. Further, the participants were assured of their anonymity and the confidentiality of the information they gave.

## 2. Healthcare at childbirth by urban poor women: Their perception and experience

People’s perceptions of health and healthcare develop according to their subjective understanding [3]. Such perceptions differ considerably between people according to their individual and aggregate socio-economic and cultural context [4]. A woman may form a perception about maternal healthcare based on her own experience or the experience of a member of her family and community, her education, the healthcare information available to her and prevailing cultural practices [5]. For example, Thai-Australian mothers perceive that a woman’s deteriorating health following a childbirth might be due to not observing the post-childbirth traditions of dietary precaution and rest as prescribed in Thai society [6]. A belief prevailing in parts of Bangladesh that post-partum bleeding is normal and necessary to cleanse the womb leads to delays in seeking treatment for abnormal haemorrhage which in turn, result in medical complications [7]. One could conceptualise a woman’s own motivation to use healthcare services based on whether she can act on her wishes [8]. However, the ability to act on one’s wishes depends to a large extent on the person’s socio-economic situation and the power of decision-making within one’s household and community. In Bangladesh society, several notions regarding childbirth practices have been persisting for generations. Even the concepts of ‘normal’ and ‘complicated’ childbirth are connected in the context of culture and societal practice [9]. The everyday experience of poor working mothers often involves performing regular household chores and working in low paid manual jobs, which leave them with little time to visit health facilities, no matter how close or convenient they may be. On the other hand, a mother’s experience of care received at a healthcare facility influences her subsequent visits there [10]. Many mothers also expressed major concerns about visiting healthcare facilities, because of costs they might incur, not in terms of the small fees they might be charged, but in terms of the hidden and undocumented costs they might have to pay as inducement to various employees to get their job done. [11]. They also expressed concerns about how to interact with healthcare providers, about maintaining their privacy during medical examinations and about the procedures they might have to follow during and after childbirth [12]. Thus, a mother’s perception and experience of childbirth and maternal healthcare leads her to adopt a certain attitude towards maternal health care–often that of avoidance, in childbirth and the post-partum period. Therefore, it may be argued that for the women of lower socio-economic households, financial cost is not the only impediment to seeking maternal healthcare at health facilities.

## 3. Materials and methods

To understand the maternal health care seeking behaviour of urban lower socio-economic class women it is important to hear their voices and understand how they articulate their perceptions and experience of maternal healthcare. Stacey (1994) points out that people themselves can best explain their experience because they are the ones who live it [13]. Their accounts do not only provide information of their experience but also go through a ‘meaning making process’ [14] in relating their experience to their social and cultural context. To achieve this, qualitative data were collected from a sample of 34 eligible women of lower socio-economic households of Dhaka by interviewing them with semi-structured questionnaires. An eligible woman is defined as someone who gave birth during the five years preceding the survey. The present sample size of 34 is based on several considerations. According to Marshall et al. [15], grounded theory qualitative studies may be based on 20 to 30 interviews and single case qualitative studies on 15 to 30 interviews. In their methods review article Baker and Edwards [16] summarise the views of experts and note that *saturation* is central to qualitative sampling, that is, “once a qualitative researcher finds that that the evidence is so repetitive that there is no need to continue to interview more participants”. In the present paper, the point of saturation was achieved after interviewing 34 women, as no new information could be obtained by adding the number of participants.

Lower socio-economic status is defined on sociological understandings of urban social stratifications, which are largely defined by ‘class’, of which income and occupation are two major components [17]. The lower socio-economic households have been chosen to represent the lower status groups, comprising workers employed in low paid unskilled and/or informal occupations.

## Study context and recruitment of research participants

Dhaka, the capital and pre-eminent city of Bangladesh is chosen for this study because in this sprawling metropolis, most of the poor live in poor quality huts in informal settlements (popularly known as slums), scattered throughout the city.

Of the 34 study participants, 23 were recruited from two slums (Sona Mia and Karwan Bazar) and 11 were recruited when they were visiting two health care centres for health checks and/or delivery. These two healthcare facilities are: Ad-Din Hospital, Mogbazar, Dhaka and an Urban Primary Health Care Centre which at that time, was managed by Marie Stopes-Bangladesh in Mohammadapur, Dhaka. Fourteen of the 23 slum dwelling participants were recruited from Sona Mia, a small slum in Mohammadpur ‘*Beribadh’* (Mohammadpur dyke) situated at the edge of Dhaka city. With an average of four members in each hut (known locally as *Jhupi*), Sona Mia has an approximate population of 1,400. This slum has no piped water or sewerage facility. The main livelihood of males of this slum is rickshaw pulling and/or working as assistants of truck drivers. Women usually work as household maids or stay at home. The women interviewed in this slum are reported to have known only three sources of maternal health care—a maternity centre of Urban Primary Health Care Project (UPHCP), the BRAC Health Centre (BHC) and the Dhaka Medical College Hospital situated a little distance away from Sona Mia.

The other nine slum participants were recruited from Karwan Bazar, a slum located near one of the largest wholesale markets of central Dhaka. Its inhabitants are better-off economically than their counterparts of Sona Mia, they are well networked, better informed about available healthcare services, more mobile and have better access to social services. However, their hygiene and housing conditions are like those of the residents of Sona Mia. Thus, these two slums represent respectively the worse off and better off slum areas of Dhaka in terms of infrastructural arrangements, employment opportunities and services. The study participants from the slums were selected by random sampling. The sampling frame for the random selection of the eligible women was prepared with the help of traditional birth attendants [known locally as *dai*].

The 11 participants recruited and interviewed at health centres were also residents of Sona Mia and Karwan Bazar who usually visited the centres to seek healthcare. These 11 participants were selected by Convenience Sampling [18] from among all those who visited the healthcare centres. The interviews were held in the local language Bangla, recorded with the consent of the participants and transcribed and translated into English by the first author. The translations were verified by persons fluent in both Bangla and English. Each interview lasted between 45 and 75 minutes. Before conducting each interview, the researcher established rapport with participants by introducing herself, not only as a researcher but also as a fellow resident of the same city and as a mother who had herself gone through a critical health condition during childbirth. The researcher also explained the objectives and purpose of the study and discussed with the participants the research ethics approval from Flinders University. As mentioned earlier, the consents of the participants to commence the interview and record the same was obtained by getting them to sign a written consent form.

## Analysis

The qualitative data collected through these interviews were analysed with the help of NVivo 10, a software program consisting of a set of tools to assist a researcher in managing data and ideas, querying and visualising data and reporting from the data in qualitative research [19]. The data were coded according to the themes and sub-themes that emerged during and after the interviews and which were important in understanding the problem being studied. This process involved careful reading and re-reading of the data ‘to remind the researchers of the breadth and depth of the content’ [20] and a step-by-step approach [21] which allowed the most significant themes to emerge. Field notes taken during the interviews were also carefully scrutinised for identifying the themes and sub-themes.

The findings of this study are presented in two main sections. The first section presents the perceptions of mothers regarding childbirth and maternal health condition that underpin their decisions whether to give birth at health facilities or elsewhere. We identify three interrelated sub-themes: “apprehension about modern healthcare facilities for childbirth”; "a culture of silence”, “childbirth is relevant only for women,: and a man cannot handle it”, and “perceived hidden costs and mistrust (of modern healthcare)”. The second section of the analysis focuses on the experience of healthcare and support received at home and healthcare facilities, showing that such experience is influenced by the status of women within households, social networks within the neighbourhood and beyond, and the relationship between health care providers and healthcare receivers (the women).

## 4. Results and discussion

The median age of the women interviewed in this study is 25 years. Their median age at first marriage is 14.5 years and that at first childbirth 16.5 years. The daily mean family income of the respondents is 305 Bangladesh Taka (equivalent to USD 4).

Regarding maternal healthcare status, 17 of the 34 women reported not having any complication or morbidity during or after childbirth. These 17 women comprise six from Sona Mia, four from Karwan Bazar and seven interviewed at health care centres. The seven women interviewed at healthcare centres visited the centres to deliver their babies. They had no existing morbidity. The other 17 women reported using antenatal care (ANC) more frequently than delivery care and postnatal care (PNC). According to these women, the quality of care at ANC was better than that during or after delivery. However, most of the ANC is reported to have been limited only to receiving tetanus toxoid (TT) injections and, for a few women, iron and vitamin tablets when healthcare workers visited the slums. In other words, the women received mostly those services which came to their doorstep.

### 4.1 Perceptions regarding childbirth and maternal health conditions

Women’s perceptions regarding childbirth and related maternal healthcare are presented under a number of subthemes—*Apprehensions about modern health facilities for childbirth*, *a culture of silence*, *childbirth as an event relevant only for women*, and *perceived hidden costs and mistrust of modern healthcare*.

#### Apprehensions about modern healthcare facilities for childbirth

Most of the women had apprehensions about hospital delivery. Such apprehensions stemmed from their negative perceptions and fears about modern health facilities such as hospitals. These comprised mostly of fears of Caesarean Section (CS) delivery and concerns about losing one’s modesty at being examined by male doctors. In general, women’s avoidance of modern healthcare facilities for childbirth can be attributed to their assumption that hospital delivery means delivery by Caesarean Sections. Even though women are provided with access cards and advice about the benefits of hospital delivery when they visit their nearest health facilities for ANC, most of them ignore such advice due to an embedded fear of CS. The reason these women are afraid of CS may be explained in the words of some of the study participants during their interviews. For example, according to Fatima:

*“At the 8*^*th*^ *month of my pregnancy*, *a health worker came to our area*, *explained to me the benefits of a hospital delivery and gave me a red card (*A red card is given to the poor pregnant women living near the Urban Primary Health Care Project Centre for free child delivery at that facility) *But I could not go to the hospital out of fear*, *because I felt that even if it should be a normal delivery*, *the doctors would intentionally go for a CS*. *We must work for a living for our whole life*, *sister [Apa]*. *If we have a baby by CS*, *we will lose several days of work*. *We will not be able to carry water or wash clothes*. *For this fear*, *I do not want to go to a hospital for delivery*. *Moreover*, *if we have one baby by CS*, *the next baby will have to be delivered by CS as well*. *It will just be unnecessary waste of money” (Fatima*, *23 years*, *Sona Mia* slum*)*

Fatima’s statement reflects the nature of a woman’s apprehension, such as mistrust of hospitals, perceived adverse physical impact of CS and its high cost. Her apprehension is largely justified by the context in which she lives. Her unwillingness to have her child delivered in a hospital is heightened by the fact that she can hardly forego her income by being absent from work for the few days that would be required for her to recover after delivering her child at a hospital.

The women who have their babies delivered at health facilities also prefer to have a normal delivery, although they may be willing to accept a CS if necessary. However, their fear of CS can still be sensed in their statements. For example, Minara came to the hospital after half a day of obstructed labour and had a CS. However, she later regretted her decision believing that if she had waited a little longer, she could have had a normal delivery.

Having a low income does not necessarily mean that these women would not go to a hospital for delivery. Some women from the Karwan Bazar slum, who have strong family and social networks, quite readily went to a hospital for delivery. Their predisposition to deliver their babies at a hospital can be explained by two factors: First, the location and structure of the slum itself is such that it is well served by various facilities, so that its residents can readily go to a hospital if the *dai*s (traditional birth attendants) are not available. Second, the positive perception of accepting the alternatives to home birth is backed up by the social network of the dwellers of Karwan Bazar. This extended social network helps the women by providing them with information and financial help and by the availability of chaperons to accompany them to the health facilities, if needed.

Women’s apprehensions about hospital-based childbirth can also come from a fear of losing one’s modesty. This is reflected in the statement of Sheher Banu:

*“We would rather die*, *but not go to the hospital for delivery*. *I have heard that male doctors do this (CS) there*. *I know*, *female doctors deliver the baby*, *but male doctors remain with them*. *Is it not a matter of shame*? *If any trouble happens*, *can a female doctor handle that*?*”* (Sheher Banu, 35 years, Sona Mia Slum)

Sheher Banu portrays the cultural orientation of shame, a woman holds in Bangladesh. Apart from these social norms related issues about modesty and honour, Sheher Banu also expressed the doubt about a female doctor’s ability to manage an obstetric complication. This reflects wider social attitudes about women’s capability of working in highly skilled professions.

#### *‘*Culture of silence’

‘Culture of silence’ has been a widely used expression in the reproductive health literature, ever since it was first coined by Mueller and Wasserheit in 1991 [22]. The propensity to keep silent about one’s own reproductive illnesses arises from the prevailing perception of ‘purity’ and ‘honour’ of women’s bodies in a society [9] and are reinforced by their inferior position within their family and society. In the present study, culture of silence is reflected in spousal communications as well as the mothers’ sense of ‘shyness’ and ‘pride’. Spousal communication on maternal health is rare among the women who participated in this study. Rehana, who reported to have been suffering from uterine prolapse, backache and anaemia, stated:

“*I told my husband about my condition*. *If I tell him more*, *he will think that “see*, *I don’t have money in my hand*, *but she keeps telling me about her problem*”. *He is male [purush jat]*, *and he might go to village or somewhere leaving us here*. *How much can I disturb him complaining about my health*? *If I cannot work properly*, *will he keep me (as his wife)*? *Will he tolerate all this*, *if I cannot get well*?*” (Rehana*, *20 years*, *Sona Mia slum)*

Some other participants of the Sona Mia slum expressed a fear that their husbands might leave them because of their ill health, therefore, they remained silent about their health conditions. Even if the women informed their husbands about their illnesses, the information would not lead to a “clarity of communication” [23] or clear and effective discussion between the spouses that could make way for treatment.

Women may also choose to remain silent due to shyness and sense of pride in having a home-based childbirth. For example, Nupur, who is suffering from post-pregnancy urinary incontinence, holds onto her shyness due to her culture, which prevents her from seeking treatment from a doctor.

According to Nupur:

*“I told my husband (about her difficulty in holding urine and burning sensation in the urinary tract)*. *He asked me to go with him to see a doctor*. *But can a woman tell this to a male doctor*? *It’s embarrassing*. *I cannot*. *I feel shy*. *I will rather see the doctor when I go to my father’s house*. *There are many people*, *my cousins*, *Aunty [khala] and many others*. *I will take any of them with me to the nearby clinic [barir kachher clinic] and go to see a doctor easily*. *There is a lady doctor [daktar Apa] there*.*”* (Nupur, 18 years,)

Nupur’s shyness worsens her condition. A similar feeling is expressed in the statement of Taslima (aged 15 years, mother of one child) of the Sona Mia slum, who does not share her problem of uterine prolapse even with her husband. Women’s shame and shyness, which are manifested in ‘silence’ about one’s own illness have been deeply rooted in the societal expectations regarding women’s reproductive roles. According to these expectations, women should have a smooth delivery without any complain. That is why, despite having modern maternal health facilities nearby, seeking clinical care for child delivery or related health conditions is sometimes not welcomed by the women and their families.

#### “Childbirth is a phenomenon relevant only to women, how can a man handle this?”

Several women interviewed during this study do not have an extended family network around them, nor do they have female friends or neighbours with whom they feel close enough because of their relatively short duration of residence in the slums. Therefore, they rely on their husbands for their needs, including the needs related to health. However, at the same time they believe that pregnancy and childbirth are purely women’s matters, which a husband cannot handle. The women even believe that a husband is not someone who can accompany them to a hospital for delivery. Husbands may also be indifferent towards their wives’ sufferings. In such situations, a birthing woman finds herself in a highly vulnerable position. In Rumu’s experience;

*“After coming from work in the morning*, *my husband found that I was suffering from pain*. *He asked me*, *“Why are you sitting like this”*? *I said*, *I think I am having pain*. *He then went to another hut and slept*.*”* (Rumu, aged 18 years)

Similarly, Nupur, also from Sona Mia slum could not go to a hospital because there was no other woman to accompany her. The ignorance and indifference of husbands, a perceivably normative behaviour, is in fact a paradox. The husband makes his wife pregnant, but he is not expected to have knowledge about pregnancy and be involved in his wife’s health and treatment. Neither the woman nor any member of her family considers the unavailability of the husband at the time of maternal health care as a matter of concern that needs to be addressed.

#### Perceived hidden costs and mistrust

Previous studies suggest that the apparently “free” services in hospitals incur many unofficial and out of pocket costs which are likely to discourage most people from utilising such healthcare services [24,25]. Almost all the women in our survey agreed that it does not matter whether the service is free or not, if someone is not able to pay any “hidden” costs, she will not receive a good service from the hospital. The following statement from Lailee (24 years) in *Sona Mia* slum expresses her anger, frustration and distrust in healthcare providers:

*“They say ‘free’*, *‘free’*, *but nothing is free*. *You even need money to fill out a form*. *They write a slip (prescription)*, *but we must buy medicine from outside*, *which is costly*, *only the doctor sees patients free of charge*. *So*, *what is free*? *That's why I cannot go*. *I don't believe that it’s free*.*”*

This eventually demotivated her from going to a hospital.

From a people’s perspective, gaps in service provision can be minimised by spending money, albeit “unofficially”. Such perceived and/or real costs of services, which are supposed to be free of charge, make hospital based maternal healthcare socially and economically burdensome for the poorer people of Bangladesh.

### 4.2 Experience of healthcare and support received at home and healthcare facilities

The study identified three key factors as contributing to the complex childbirth experience of the women—(i) their status within their households, (ii) their social network and (iii) the relations between healthcare providers and receivers.

#### Status of the women within their households

The women interviewed in this study have less than primary school education (Year 5). Some of them work for pay, but their earnings are very low. In most cases their entire income is spent on supporting their households, but a regular income, even if low, empowers them to have a say about health care and prepares them for obstetric emergencies. It also helps them to make financial plans for pregnancy and delivery. Robena (aged 22 years), interviewed at Ad-din Hospital, works in a garment factory. She has saved an amount equivalent to her income that she would lose during her eventual stay in a hospital for childbirth and post-partum period. Robena has been able to do this, because she earns and spends her own money. It is evident from the interviews that the women who are employed have more control over decision making on household issues as well as on their own healthcare. This finding is consistent with other studies conducted in urban and rural areas. [26,27]

#### Social network in the neighbourhood and beyond

In the absence of health service provision and appropriate health care information, social and community networks in the slums provide the needed support and care for the women. Those who do not have such social and family support, find it much more difficult to have proper maternal healthcare. To quote Rahana:

*“My husband asked me*, *‘please go and see if you can borrow some money from somebody*. *Then we can see a doctor’*. *But I could not get money from anybody*. *In this yet a strange place*, *who will give money to us*? *That’s why I could not go for treatment*. *I am staying like this”*. (Rahana, 20 years,).

On the other hand, Jhumur (aged 28 years, mother of one child) of Karwan Bazar slum was able to pay for her blood transfusion during delivery mainly by borrowing money from neighbours. Jhumur was born, brought up and married in the same slum, a fact which gave Jhumur and her husband a great advantage in terms of social and community networking.

**Fig 1:**
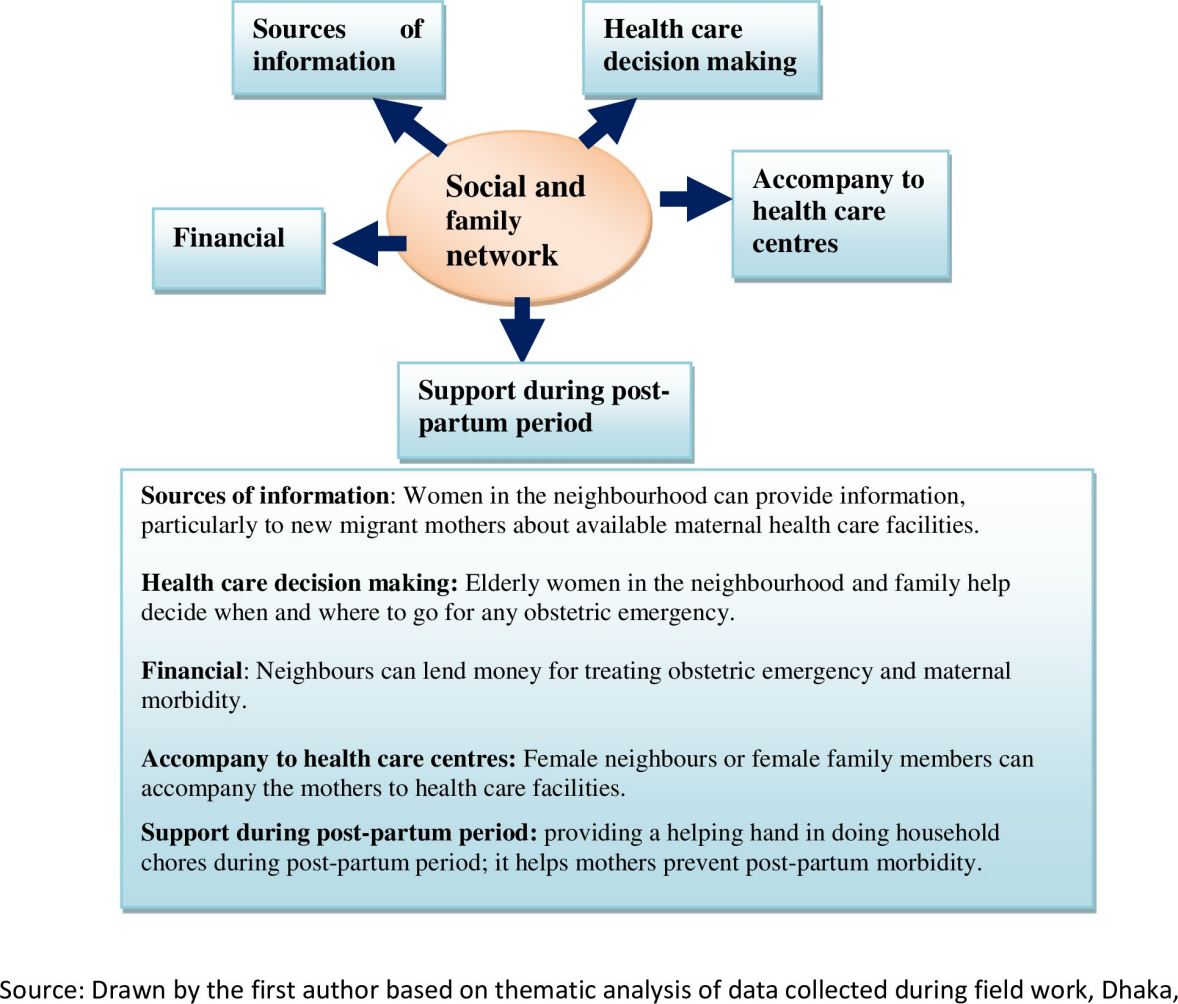
Support that social and neighbourhood networks provide.

Fig 1 illustrates how social and family networks provide help with maternal healthcare in the slums in various important ways. They help the women in accessing maternal healthcare and provide them with physical, financial and moral support during and after delivery.

Duration of residence in an urban location is a crucial factor for building a social and neighbourhood network. Recent migrants to an urban area, such as Rehana cannot access to an extended family network which they left behind in their villages. The absence of family members, inadequate social acquaintances within the neighbourhood and financial insecurity make it difficult for such women to get the kinds of support shown in Fig 1. On the other hand, those who migrated to Dhaka long ago already have a local social and family network and have become familiar with the available maternal healthcare services, their benefits and challenges.

#### Experience at the hospital: Relations between healthcare providers and healthcare receivers

While the root of many apprehensions can be traced back to the experiences of their relatives, neighbours and friends some of the women narrated difficulties they experienced themselves at healthcare facilities. Marjia from the Karwan Bazar slum described her ANC experience in which the interpersonal communication and the behaviour of the doctor discouraged her from going back there. She explains:

*“I went to Maternity Centre (of UPHCP) only twice*. *I had my check-up and took vitamins*. *I did not go there anymore*. *And I did not like the doctor who replaced my previous doctor*. *I do not like the way she talks [Tar kotha amar pochhondo hoyna]*. *The new doctor is rude*. *She does not care much about the patients; she wants to do everything fast*. *She does not try to speak to us and make us understand things*. *That's why we don't go*.*”* (Marjia, 24 years)

The above quote from Marjia clearly indicates what a woman expects to receive from her healthcare provider. She wants to be treated nicely and respectfully and get the things and information explained to her clearly. But when she found that her expectation was not fulfilled, she rejected the thought of going there again. This is that kind of situation, where, according to Bruce [28] ‘interpersonal relations’ go beyond providing accurate information and the degree at which it is comprehended by service receivers. The desired outcome of interpersonal relations between the health care providers and receivers may be “that the client reports a belief in the competence of the provider, trust of a personal nature and a willingness to make contact again themselves or even refer others”. It has been decades since Bruce [28] made this observation, but there appears to be no improvement in inter-personal skills of healthcare providers.

The findings and discussion presented above show that the perception and understanding of maternal healthcare, childbirth and the post-partum period by women of lower socioeconomic status are shaped by their cultural construction of childbirth, together with inadequate knowledge about modern childbirth at a health facility assisted by medically qualified birth attendants, extent of their urban social network and adverse socio-economic condition for affording hospital based childbirth. Their perception and understanding are in stark contrast to those of women of upper socio-economic status in Dhaka city, which were also examined in the larger study conducted by the first author for her PhD dissertation [29]. The upper socio-economic status women of Dhaka have apprehensions about natural childbirth at home with labour pain and therefore they prefer medicalised childbirth in a hospital and choose to have a Caesarean Section (CS) delivery even when not medically required. These women’s affluence makes it affordable for them to have the luxury and rest available at private hospitals and to have social and family support to do household chores and other works [29]. The contrast in the perception, understanding and practice of maternal healthcare and childbirth between the lower and upper socio-economic status women can be exemplified by the percentage of CS delivery, which is 61.3% for the richer women but only 13% for the poorer women [30]. This difference in maternal health care services and childbirth practised by the Bangladesh women clearly indicates that childbirth care is influenced by a woman’s socio-economic status and her cultural perception of childbirth.

## 5. Conclusion

The perception and experience of women interviewed in this study reveal patterns of health care behaviour which can be broadly described as a practice of avoidance of facility based maternal health care. The findings of this study suggest that women of urban slums of Dhaka have a deeply rooted fear of medical intervention in childbirth for many different perceived and practical reasons, which include unfamiliarity with the treatment and institutional processes, unofficial or hidden costs, lack of social and family network support within their neighbourhood, concept of honour and shame *[sharam]*, a culture of silence and inadequate spousal communication on health issues. As a result, even though low cost health care facilities are within their reach geographically, the mothers or would be mothers and their families are reluctant to utilise such facilities to deliver their babies.

Moreover, the financial, physical and social limitations of the women and those of their family members prevent them from seeking maternal health care at health facilities. They do not make any effort to overcome these barriers because they appear to be content with their age-old perception of pregnancy and childbirth as part of nature and not a special event worth giving any special attention to. Therefore, most women tend to remain unenthusiastic about facility based maternal healthcare.

*“Those are not for poor people like us*. *Even if I agree that they give free service*, *we cannot manage to go there*. *We must arrange our daily*, *three meals a day [tin bela khawa]*, *we must look after our children*, *give service to husband [shami r sebajotno]*. *When can I go there (health facilities)*, *stand in line and so on*. *Rather*, *whatever is in store for us*, *will happen*. *What’s the benefit of worrying too much*?” (Laila, 24 years, Sona Mia slum)

Laila’s statement indicates that her reluctance to go to a hospital is borne out of her experience of living in poverty, continuous hard work to earn a living and maintaining her family.

The disinclination of the women to deliver their babies at a health facility does not necessarily mean that they are opposed to using modern healthcare for childbirth. Their predisposition against using healthcare facilities may be explained by the apprehensions they have developed through their life experience and perception.

Bangladesh has expanded its maternal health service by setting up low cost government and non-government hospitals at different levels all over the country so that women can reach them conveniently, free of charge or at low cost. The government has also expanded ANC coverage throughout the country. Consequently, there has been a remarkable reduction in fertility and maternal mortality in Bangladesh. However, several studies [31] suggest that childbirth is generally considered as less of a health-related event for the women and more of a social and family event that needs a greater understanding of women’s beliefs, perceptions and apprehensions about pregnancy care and childbirth at modern healthcare facilities, and not just an increased availability of low cost maternal health care facilities in physical proximity of the women. Therefore, it would be crucial to provide these women with information to help them break free of their perceived fear of hospital based childbirth, address the issues of hidden costs of visiting hospitals, and take steps to improve culturally sensitive interpersonal relations between healthcare service providers and the women seeking such services.
